# The loss of glycoprotein nonmetastatic melanoma protein B (GPNMB) alters endothelial cell permeability, metabolism, and survival during infectious challenge

**DOI:** 10.1042/CS20256682

**Published:** 2026-01-14

**Authors:** Milene T. Fontes, Celia K. Lamb, Allison Pourquoi, Cassandra Lauren Atzrodt, Hong-Ngan Nguyen, Sourav Panja, Ryan Jordan Stark

**Affiliations:** 1 Division of Pediatric Critical Care Medicine, Vanderbilt University Medical Center, Nashville, U.S.A.

**Keywords:** endothelial cells, GPNMB, inflammation, metabolism, osteoactivin

## Abstract

During severe systemic infections, also known as sepsis, excessive cytokines and reactive oxygen species lead to endothelial dysfunction. Glycoprotein nonmetastatic melanoma protein B (GPNMB) has been implicated in regulating cellular functions, particularly within the vasculature during inflammation, but its effect on infection-mediated endothelial injury remains unclear. Data obtained from the Gene Expression Omnibus (GEO) show that GPNMB expression is systemically reduced following an infectious challenge. Therefore, to investigate the role of GPNMB during infection-mediated endothelial inflammation, we utilized human microvascular endothelial cells (HMVECs) with or without GPNMB knockdown (siGPNMB) and exposed them to heat-killed *Escherichia coli* (HKEC), one of the most common pathogens associated with sepsis. Silencing GPNMB altered the expression of 1453 genes via RNA sequencing, affecting cytoskeleton function and the response to stimuli. When assessing the endothelial monolayer under basal conditions, siGPNMB cells displayed higher transendothelial electrical resistance (TEER), consistent with RNA sequencing pathway analysis, but exposure to HKEC resulted in increased barrier dysfunction compared with controls. Furthermore, compared with controls, assessments of viability, proliferation, and migration were compromised in siGPNMB cells following HKEC exposure. Exposure to HKEC decreased the oxygen consumption rate in controls and increased the extracellular acidification rate, but neither was changed in siGPNMB cells, indicating impaired metabolic adaptation and further corroborating aspects of the RNA sequencing data. Our findings demonstrate that GPNMB reduction hinders the endothelial response to infectious stimuli, resulting in decreased metabolic fluxes and dysfunctional endothelium during infectious challenges.

## Introduction

Endothelial cells, which act as the interface between blood and tissue, have long been recognized as crucial regulators of inflammation, particularly in serious systemic infections referred to as sepsis [1–4]. When encountering an infectious stimulus, endothelial cells play a crucial role in the innate immune response by facilitating leukocyte adhesion and transmigration and releasing inflammatory mediators to recruit immune cells and combat the pathogen. Further, the tight monolayer they form under resting conditions becomes permeable, allowing immune cells to access affected tissues [5,6]. However, these beneficial effects can be negated by excessive inflammation and oxidative stress, resulting in increased permeability, apoptosis, and coagulation dysregulation, which culminates in endothelial dysfunction and vascular instability [7–10]. The observed dysfunction is often the result of alterations in metabolism and energy needs to promote endothelial survival during systemic stress [11]. While multiple pathways may contribute to the disrupted endothelial phenotype, recent evidence highlights an immune-mediated role of glycoprotein nonmetastatic melanoma protein B (GPNMB) in regulating cell differentiation, migration, inflammation, and metabolism [12,13].

GPNMB is a highly glycosylated type I transmembrane glycoprotein that can also signal as a cleaved, soluble fraction. It is expressed in various tissues and cell types [12,14]. Studies have suggested that the extracellular domain of GPNMB, also known as osteoactivin, may act as a chemoattractant, promoting angiogenesis and the recruitment of endothelial cells [15]. In a model of myocardial infarction, cardiac injury was enhanced during GPNMB loss, suggesting a protective effect [16,17]. Although few studies have evaluated the role of GPNMB on vascular function, one study observed impairment of both acetylcholine relaxation and blood flow recovery in the iliac arteries of GPNMB knockout mice, demonstrating a relationship between endothelial function and the GPNMB abundance [18]. Alternatively, in another paper, the reduction of GPNMB improved vascular inflammation in a model of diet-induced atherosclerosis, suggesting an unclear role for GPNMB in regulating the vasculature [19].

Recognizing the significant role of endothelial dysfunction in sepsis and the emerging importance of GPNMB in regulating vascular responses, we aimed to explore how endothelial GPNMB influences inflammation and metabolism during bacterial infection. To examine this, we analyzed gene expression data for GPNMB obtained from peripheral blood mononuclear cells (PBMCs) of septic patients or whole blood exposed to *Escherichia coli* and *Staphylococcus aureus* in the Gene Expression Omnibus (GEO) database. We observed a significant reduction in GPNMB levels in septic shock and bacterial challenge. To further understand the implications of this reduction, we used knockdown models to assess the impact of reduced GPNMB expression in endothelial cells during an infectious challenge. We specifically hypothesized that reduced GPNMB expression exacerbates inflammatory responses, impairs cellular repair mechanisms, and contributes to endothelial dysfunction. To test this hypothesis, we genetically modified GPNMB expression in endothelial cells and subjected them to a bacterial challenge with *Escherichia coli* (*E. coli*), a common sepsis-associated pathogen. Through this approach, we aimed to elucidate the role of GPNMB in endothelial inflammation and metabolism, potentially identifying GPNMB as a therapeutic target to mitigate endothelial dysfunction.

## Methods

### Human transcript profiles

Gene array profiles of blood samples were obtained from the publicly available National Centers for Biotechnology Information (NCBI) Gene Expression Omnibus (GEO) database. Values were obtained from GEO Dataset GSE48080 initially collected by Severino et al. ‘Gene expression in peripheral mononuclear cells from septic patients secondary to community-acquired pneumonia: patterns of gene expression and outcomes’ [20] and GSE237960 collected by Stark et al. ‘Differential signaling effects of Escherichia coli and Staphylococcus aureus in human whole blood indicate distinct regulation of the NRF2 Pathway’ [21]. Datasets were queried for GPNMB from samples run on an Agilent-014850 Whole Human Genome Microarray and Illumina NovaSeq 6000, respectively.

## Endothelial cells and culture conditions

Human dermal microvascular endothelial cells (HMVEC) were obtained from the American Type Culture Collection (ATCC CRL-4060; Manassas, VA, U.S.A.). The cells were cultured in Vascular Cell Basal Medium (ATCC; PCS-100–030), supplemented with Microvascular Endothelial Cell Growth Kit-VEGF (ATCC; PCS-110–041), penicillin (100  U/ml), and streptomycin (100  U/ml), at 37°C in a humidified 5% CO2 atmosphere.

## Cell transfection and stimulation

According to the manufacturer’s recommendations, HMVECs were treated with siRNA [scrambled siControl (D-001810–10-05), siGPNMB (L-040019–01-0005)]. In brief, siRNA was procured from Dharmacon (Revvity Inc., Waltham, MA, U.S.A.). siRNA (25  nmol/l) was incubated with Dharmafect (Dharmacon; T-2001–01) in a serum-free medium for 20  min. The resultant complex of siRNA-Dharmafect was added to the cells in 5% FBS media without antibiotics for 6  h. Afterward, the transfection media was replaced with complete media, including antibiotics, for 72  h siRNA, according to the manufacturer’s guidelines. Transfection efficiency was detected for western blot analysis (Supplementary Figure S1). The cells were stimulated for 6 h in the presence or absence of 3 × 10^8^ cells/ml of *heat-killed E. coli* (tlrl-hkeb2; InvivoGen, San Diego, CA, U.S.A.), or, 100 ng/ml *E. coli* lipopolysaccharide (MilliporeSigma, Burlington, MA, U.S.A.).

## RNA isolation, sequencing, and analysis

Total RNA was isolated from siRNA-treated HMVECs using the Qiagen RNeasy Mini Kit. The extracted RNA was assessed for quality at the Vanderbilt Technologies for Advanced Genomics (VANTAGE) core, ensuring sufficient RNA concentration, a 260/280 ratio of 2.0, and a high RNA Integrity Number (RIN, 9.7–10 for samples), as determined by an Agilent Bioanalyzer (Agilent Technologies Inc., Santa Clara, CA, U.S.A.). Poly(A) RNA sequencing was conducted at the VANTAGE core using the Illumina NovaSeq 6000 platform (Illumina Inc., U.S.A.) with paired-end 150 sequencing, generating approximately 60 million PF reads per sample. Raw sequencing reads (FASTQ files) were uploaded to the Partek Flow server for pre-alignment quality assessment (Partek Inc., Illumina, San Diego, CA, U.S.A.). Reads were aligned to the human genome (Hg38 assembly) using STAR 2.7.8 a, and gene-level quantification was performed against Ensembl transcript annotations (version 93) using Partek’s expectation–maximization (E/M) annotation model. Gene counts were normalized to fragments per kilobase of exon per million mapped fragments (FPKM) per sample and subsequently log-transformed with an offset of 0.0001. Hierarchical clustering and principal component analysis (PCA) were conducted on the normalized, log-transformed data using Partek Genomics Suite. Differential gene expression analysis was visualized using volcano plots, generated through the online tool VolcaNoseR (https://huygens.science.uva.nl/VolcaNoseR), with a fold change threshold of ≥ 1.5 and *P* ≤ 0.05. Pathway enrichment analysis was performed using the Gene Ontology (GO) Biological Process database via the Enrichr platform (https://maayanlab.cloud/Enrichr/#).

## Cell viability and proliferation assay

Viability and proliferation assays were performed using the Cell Counting Kit-8 (K1018, APExBIO, Houston, TX, U.S.A.). 72 h after the RNA sequencing protocol, cells were seeded in a 96-well plate, 100  μl cell suspensions (5 × 10^4^) with 5% FCS medium was added to a 96-well plate and received a 6 h stimulation with HKEC (3 × 10^8^ cells/ml) at 37°C, as described above. In additional experiments, control cells were exposed to different concentrations (10, 25, and 50 ng/ml) of Recombinant Human GPNMB Protein (11305-H08H; Sino Biological) [22] . Then, 10  μl CCK-8 reagent was added to each well, cells were incubated for 2  h at 37°C, and the first optical density at 450  nm was measured (FLUOstar Microplate Reader). Measurements were repeated every 12 h for the proliferation experiment, and at the end of 24 h for the cell viability experiment. The growth rate was expressed as a value relative to hour 0.

## Endothelial cell migration assay

After the RNA sequencing protocol, HMVECs were seeded at 5 × 10^5^ cells/well in 5% FBS medium and received a 6 h stimulation with HKEC (3 × 10^8^ cells/ml) at 37°C as described above. Afterward, cells were washed with phosphate-buffered saline, and 1% FBS medium cell culture was added. A 200 µL pipette tip made a single vertical wound through the cell monolayer in each well. The medium (1% FBS) was replaced, and the plates were returned to the incubator. Three images of each well (top, middle, and bottom) were captured under a phase-contrast microscope (4× objective) (Olympus IX70) at different time points (0, 6, 12, and 18 h). HMVEC migration was plotted by calculating the percentage of the original wound area covered by migrating cells at each time point using ImageJ (National Institutes of Health, U.S.A.).

## Transendothelial electrical resistance (TEER) assay

At 60 h post-siRNA exposure, HMVEC (siGPNMB and control cells) were plated on the CellZScope and allowed to settle for 12  h to achieve confluence and complete 72  h siRNA incubation time before HKEC exposure. The determination of TEER was performed using the CellZScope2 (nanoAnalytics GmbH, Münster, Germany). For assays involving siRNA, siRNA treatment was done prior to plating cells to reduce the impact of washing on monolayer formation and resistance. HMVECs were coated on ThinWell Cell Culture inserts (0.4  μm pore diameter, Greiner Bio-One, Monroe, NC, U.S.A.) using a previously standardized protocol [23–25]. Cells were then plated at 40,000 cells/well and allowed to grow to confluence over 12  h. Afterward, they were stimulated with HKEC (3 × 10^8^ cells/ml) or LPS (100 ng/ml) and examined for TEER.

## Enzyme-linked immunosorbent assay (ELISA)

Media from the treated HMVEC was collected after a 6 h stimulation with HKEC. Concentrations of secreted Human IL-8/CXCL8 (DY208; R&D System), Human TNF-alpha (DY210; R&D Systems) and TIMP-1 (DY970; R&D System) were assessed following the guidelines of the manufacturers. The optical density was measured at 450 nm (FLUOstar Microplate Reader, BMG Labtech, Cary, NC, U.S.A.), and the secreted protein concentrations were analyzed using a standard curve.

## MMP activity assay

Matrix metalloproteinase (MMP) activity was measured using the Amplite Universal Fluorometric MMP Activity Assay Kit (AAT Bioquest, #13510, Sunnyvale, California, U.S.A.) in the supernatants from treated HMVEC (siGPNMB and control cells), which were collected after a 6 h stimulation with HKEC. Following the kit instructions, 25 μl of each test sample was added to a solid black 96-well plate, along with 25 μl of the supplied assay buffer. The samples were incubated for different times with 4-Aminophenylmercuric Acetate to promote MMP activation. Activation was performed for MMP-2 (gelatinase), MMP-3 (stromelysin), and MMP-9 (gelatinase). Then, 50 μl of the MMP Green working solution was added to each well. The plate was then incubated for 30 min at 37°C in the dark, with mixing. Fluorescence intensity was measured every five minutes at Ex/Em = 485/535 nm using a FLUOstar Microplate Reader (BMG Labtech).

## Western blot analysis

Immediately after completion of the stimulation period with vehicle, HKEC, or LPS, the cells were washed with PBS and then collected and lysed. Protein extracts (50  μg/sample) were separated by SDS electrophoresis on a polyacrylamide gel (8% and 12%) and transferred to nitrocellulose membranes. Membranes were blocked with Odyssey Blocking Buffer (LI-COR Biosciences, Lincoln, NE, U.S.A.) for 1  h at room temperature. Membranes were incubated with primary antibodies overnight at 4°C on a rocker. The list of antibodies used is listed in Supplementary Table S1. Afterward, membranes were incubated with fluorescent secondary antibodies and analyzed using the Odyssey Imaging System (LI-COR Biosciences). Protein quantification was performed via densitometry and normalized as a ratio of expressed protein to α-Tubulin, α-Actin, or phosphorylated protein to respective total protein.

## Mitochondrial oxygen consumption and glycolysis assay

HMVECs were plated in a 96-well Seahorse assay plate at 15,000 cells/well in Seahorse Assay Media and assessed on the Seahorse XFe 96 Extracellular Flux Analyzer (Agilent Technologies, Santa Clara, CA, U.S.A.). A glycolysis stress test was performed in HMVECs by sequentially treating them with 10 mM glucose (RPI, Mount Prospect, IL, U.S.A.), 1 mM oligomycin (Agilent Technologies), and 50 mM 2-deoxyglucose (2-DG) (MilliporeSigma). Mitochondrial stress was performed in assay media supplemented with 10 mM glucose, and cells were sequentially treated with 1 mM oligomycin (Agilent Technologies), 1 mM FCCP (Agilent Technologies), and 0.5 mM of antimycin A and rotenone (Agilent Technologies).

## ADP/ATP ratio assay

HMVECs SCR and siGPNMB were plated in a 96-well plate. Immediately after the vehicle or HKEC stimulation period was completed, the ATP/ADP ratio was measured using the ADP/ATP Ratio Assay Kit according to the manufacturer’s instructions (MAK135; MilliporeSigma).

## Rescue experiment

To establish the causality between the presence of the protein and its role in inflammatory processes, HMVECs were transfected with siRNA for SCR or siGPNMB (protocol described above) followed by reintroduction through the administration of Recombinant Human GPNMB Protein (11305-H08H; Sino Biological) at a concentration of 25 ng/ml for 24 h before exposure to the inflammatory challenge (HKEC 3 × 10^8^ cells/ml for 6 h). Subsequently, the cells were washed with PBS and then collected and lysed for evaluation of protein expression.

## Statistical analysis

Values are mean ± standard error of the mean (SEM), and ‘n’ represents the number of independently performed experiments in cultured cells. Graphs were generated using GraphPad Prism 9.0 (GraphPad Software, San Diego, CA). Student’s t-test, one or two-way ANOVA assessed statistical differences for parametric endpoints. Post hoc comparisons were performed using Tukey analysis to compare all groups. For nonparametric clinical analysis, a Kruskal–Wallis test with Dunn’s correction was used. A *P-value* of < 0.05 was considered statistically significant.

## Results

### GPNMB is down-regulated in acute infectious conditions

**Figure 1:**
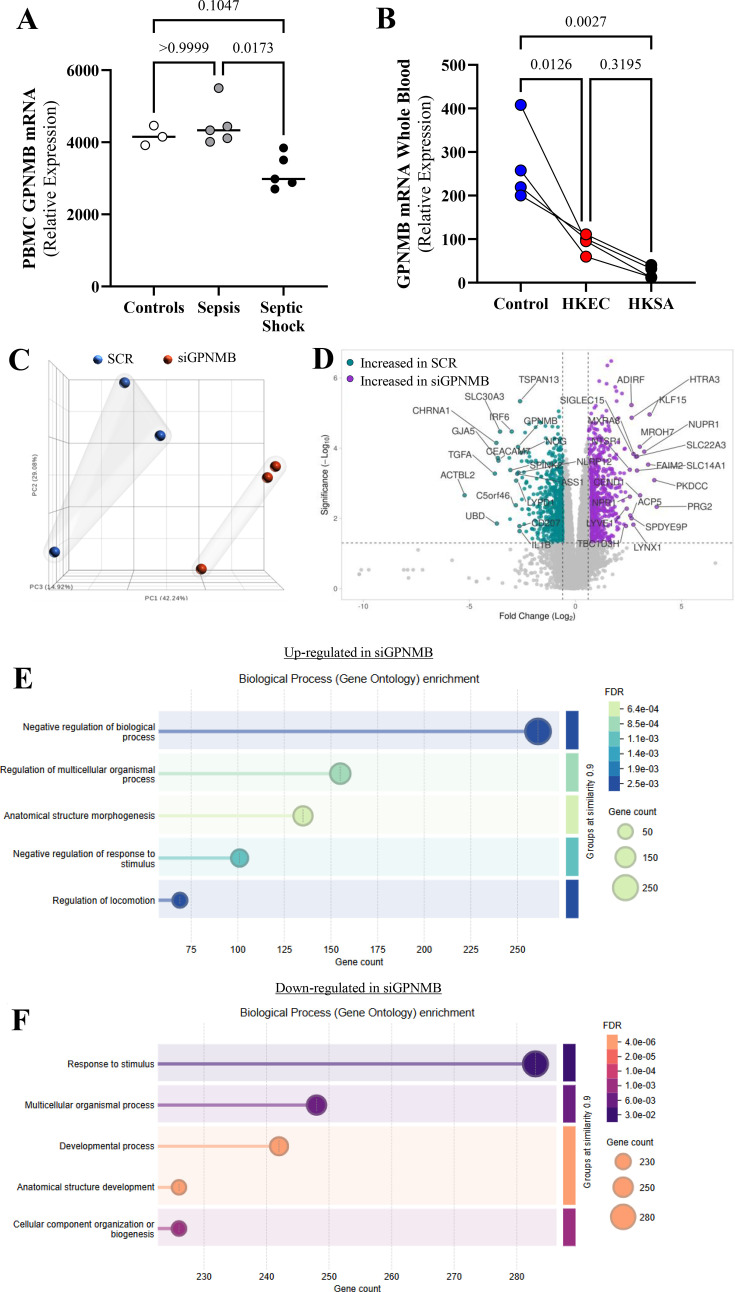
GPBMB expression analysis of GEO datasets and RNA-seq data from human microvascular endothelial cells (HMVEC) treated with scrambled siRNA (SCR) or GPNMB-targeting siRNA (siGPNMB). (**A**) GPNMB gene expression in peripheral blood mononuclear cells (PBMC; 1 × 10^7^ cells/ml) from healthy patients or day 0 (presentation to the hospital) in patients identified as having sepsis or septic shock. (**B**) GPNMB gene expression in unstimulated human blood or paired samples stimulated with heat-killed E. coli (HKEC, 3 × 10^8^ cells/ml) or S. aureus (HKSA, 3 × 10^8^ cells/ml) for 6 hours. Each dot represents an individual sample. Patient-derived transcript data are presented as medians, and paired *ex vivo* stimulated samples are shown as a spaghetti plot. Statistical analysis: Kruskal–Wallis with Dunn correction for nonparametric comparisons and one-way ANOVA with Tukey correction for parametric comparisons. (**C**) Principal component analysis (PCA) plot depicting the clustering of HMVECs based on treatment groups, with SCR-treated cells in blue and siGPNMB-treated cells in red. (**D**) Volcano plot showing differentially expressed genes between SCR and siGPNMB treatments. Genes significantly up-regulated in SCR (log2 fold change > 1.5, *P* value < 0.05) are highlighted in green, and those up-regulated in siGPNMB (log2 fold change < −1.5, *P* value < 0.05) are highlighted in purple. Nonsignificant genes are gray. (**E-F**) Bar plot illustrating the top 10 enriched GO terms in biological processes for differentially expressed genes, five up-regulated (**E**) and five down-regulated (**F**) by GPNMB knockout. Bars represent fold enrichment, with color intensity indicating the false discovery rate (FDR).

Although previous data in the literature support that GPNMB may mediate inflammatory mechanisms, original studies showing how GPNMB behaves in the face of an inflammatory response are lacking and need further understanding. To assess whether GPNMB is altered in humans in the presence of severe infections, we utilized the Gene Expression Omnibus (GEO) database. In the first dataset, we compared the gene expression levels of GPNMB in PBMCs from patients with sepsis or septic shock (GSE48080), compared with healthy controls. In that cohort, GPNMB mRNA levels were lowest in patients who exhibited septic shock, which is clinically associated with worse vascular dysfunction (Figure 1A) [26]. Next, utilizing prior whole blood RNA sequencing data we had published, we examined the transcription of GPNMB in unstimulated (control) human blood to paired blood exposed to heat-killed *E. coli* (HKEC) or S. aureus (HKSA), two of the most common pathogens in sepsis and severe infections [21]. Again, we saw a significant reduction in GPNMB transcription across all samples after stimulation with both types of bacteria (Figure 1B), suggesting that common bacterial pathogens knock down GPNMB transcription and the level of suppression is associated with the severity of vascular illness.

### GPNMB knockdown in resting endothelial cells is anti-inflammatory

Recognizing the critical role of endothelial cells during an infectious challenge and that severe infections suppress GPNMB transcription, we aimed to investigate the impact of reduced GPNMB levels in these cells. Previous studies on endothelial cells have primarily focused on their functions from different perspectives, such as vascular senescence [18,27]. To obtain a baseline understanding of the role of GPNMB in endothelial cells under resting conditions, we used siRNA followed by RNA sequencing to explore the transcriptomic landscape. GPNMB knockdown altered the expression of 1453 genes (813 up-regulated and 640 down-regulated). Principal component analysis (PCA) demonstrated the separation of the transcriptomic profiles of HMVECs exposed to scrambled siRNA (SCR) compared with those with siGPNMB (Figure 1C). Figure 1D shows the individual genes that changed, with PRG2, a regulator of anti-microbial activity, being the most up-regulated gene by fold change following GPNMB reduction, and TGFA, a growth factor that plays a significant role in the regulation of cell proliferation, differentiation, and development, being the most down-regulated gene in GPNMB knockdown cells [28–30]. When examining the overall biological processes related to transcriptomic changes induced by GPNMB knockdown that had a false discovery rate (FDR) of <0.05, the most altered pathways were related to cellular morphology, structure, and the response to stimulation (Figure 1E and F).

**Figure 2:**
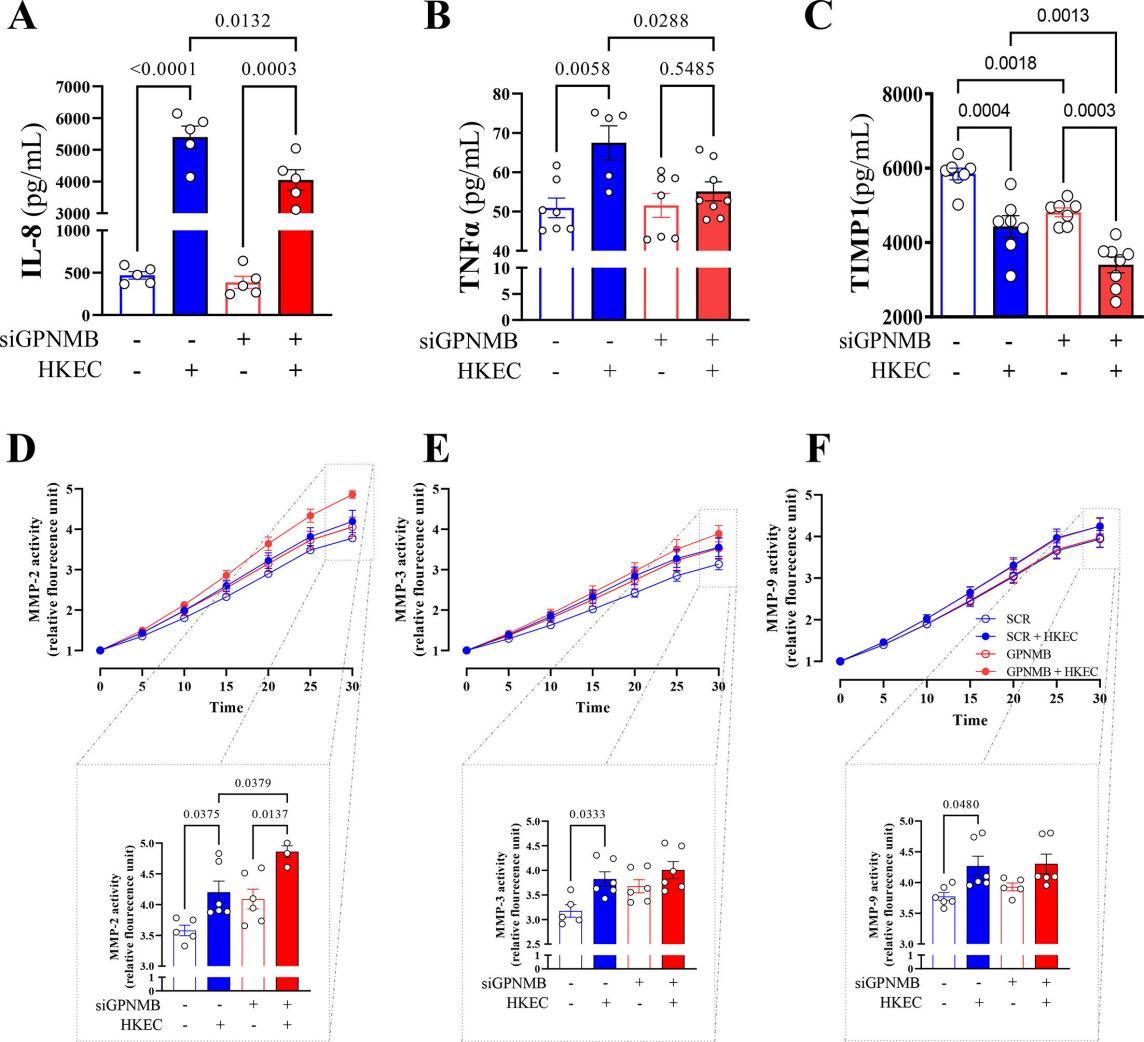
Cytokines, the tissue inhibitors of metalloproteinases (TIMPs) and metalloproteinases release in GPNMB-knockdown (siGPNMB) and scrambled control (SCR) HMVECs upon inflammatory challenge. **(A**) Interleukin-8 (IL-8), (**B**) tumor necrosis factor-alpha (TNF-α), (**C**) the tissue inhibitors of metalloproteinases 1 (TIMP1) concentrations, and (**D**) metalloproteinases-2, (**E**) metalloproteinases-3, and (**F**) metalloproteinases-9 activity in the culture medium of human microvascular endothelial cells (HMVECs) treated with scrambled siRNA (SCR) or GPNMB-targeting siRNA (siGPNMB), following stimulation with either vehicle or heat-killed *E. coli* (HKEC, 3 × 10⁸ cells/ml) for 6 h. Each dot represents an individual sample. data are presented as mean ± SEM. Statistical analysis: Two-way ANOVA, followed by the Tukey post hoc test.

Given this, to assess the general inflammatory cytokine output of endothelial cells to the clinically relevant stress of bacterial exposure, such as seen in sepsis, endothelial cells were exposed to siRNA to GPNMB or scrambled siRNA and then challenged with heat-killed *E. coli* (3 × 10^8^ cells/ml, HKEC) for 6 h. The concentration of interleukin (IL)-8 and tumor necrosis factor (TNF)-α, important regulators of the response to inflammatory diseases, was assessed [31,32]. Both increased compared with unstimulated conditions in the presence of HKEC for cells with or without reduced GPNMB (Figure 2A and B). However, when compared across siRNA-treated groups, IL-8 and TNFα after HKEC exposure were lower in siGPNMB cells than in SCR controls. The presence of HKEC also reduced tissue inhibitors of metalloproteases (TIMP-1) in both siRNA-treated groups; however, in siGPNMB cells, the reduction was enhanced owing to a lower level of TIMP-1 expression under resting conditions (Figure 2C). Supporting the data observed with the MMP inhibitor present, HKEC significantly increased the activity of MMP-2 (Figure 2D), MMP-3 (Figure 2E), and MMP-9 (Figure 2F) in SCR cells. Although in siGPNMB cells, HKEC only increased MMP-2 (Figure 2D), the activity was higher than both in the absence of HKEC and in SCR cells stimulated with HKEC. Although MMP-3 and MMP-9 tended to increase their activity, there were no significant differences between the conditions of siGPNMB cells (Figure 2E and F).

### GPNMB loss regulates endothelial barrier function differentially during infection

**Figure 3:**
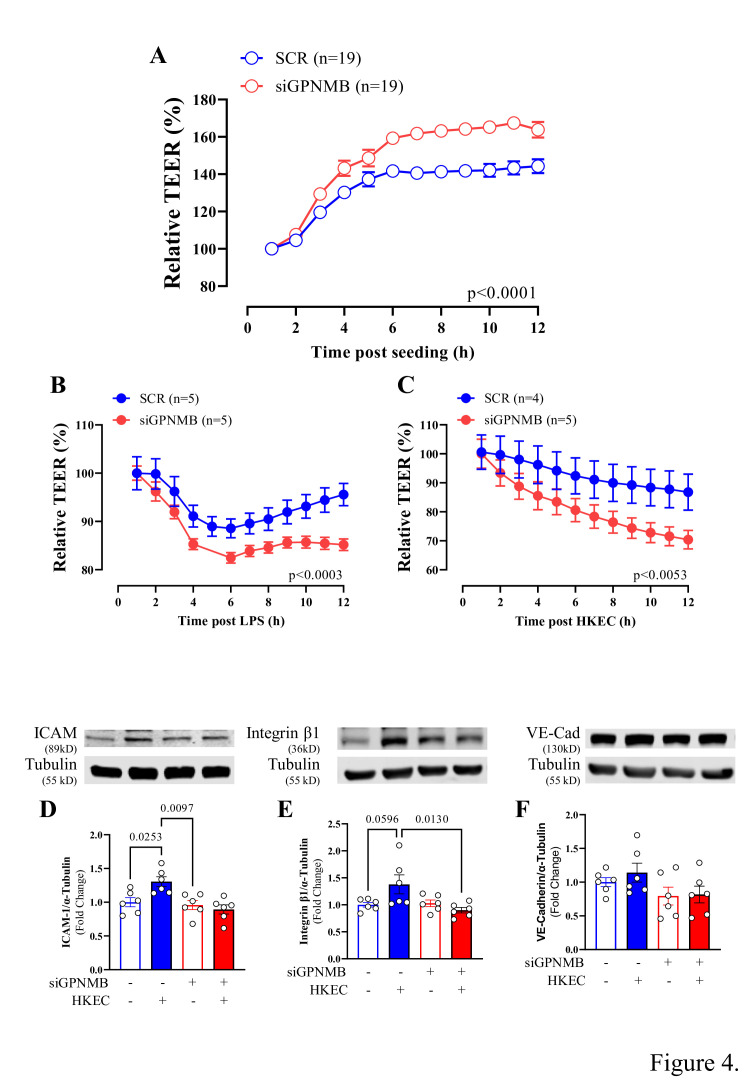
Endothelial barrier function and protein expression in GPNMB-knockdown and scrambled HMVECs. **(A**) Transendothelial electrical resistance (TEER) of human microvascular endothelial cells (HMVECs) treated with scrambled siRNA (siControl) or GPNMB-targeting siRNA (siGPNMB). (**B**) TEER measurements following lipopolysaccharide (LPS, 100 ng/ml) exposure, normalized to baseline values prior to stimulation. (**C**) TEER measurements after exposure to heat-killed *E. coli* (HKEC, 3 × 10⁸ cells/ml), normalized to baseline values. (**D-F**) Protein expression levels of ICAM, Integrin-β, and VE-Cadherin, with α-Tubulin used as a loading control. The number of samples used in each experiment (***n***) is expressed in parentheses or dots. Data are presented as mean ± SEM. Statistical analysis: Two-way ANOVA, followed by the Tukey post hoc test.

Since we found that GPNMB knockdown altered the expression of genes related to regulating endothelial morphology and the cytoskeleton, including TIMP-1, which has been shown to regulate the stability of cell-cell junctions between endothelial cells [33], we performed trans-endothelial electrical resistance (TEER) assays as a surrogate of measuring monolayer integrity that is often perturbed during sepsis [34,35]. Prior to inflammatory stimuli, in cells in which GPNMB was reduced, increased monolayer resistance was observed compared with control cells (Figure 3A). However, in the presence of HKEC (Figure 3A) or its primary toxin, lipopolysaccharide (LPS, 100 ng/ml, Figure 3B), ECs exhibited significantly lower resistance. This reduction was more pronounced in siGPNMB cells, indicating that while siGPNMB may initially strengthen the barrier, its absence makes the endothelial cells more vulnerable to barrier disruption during inflammatory challenges. To examine if HKEC altered adhesion-associated proteins in siGPNMB cells, we performed protein analysis on the expression of intercellular adhesion molecule 1 (ICAM) and integrin β1. HKEC increased the expression of adhesion molecules in SCR cells (Figure 3D and E; Supplementary Figure S4B-4E). This effect, however, was absent in siGPNMB cells, suggesting that GPNMB is essential for up-regulating adhesion molecules in response to HKEC-induced inflammation. Conversely, global VE-Cadherin expression (Figure 3F; Supplementary Figure S4F) was not altered by the knockdown of GPNMB or stimulation with HKEC. We analyzed the expression of zonula occludens-1 (ZO-1) and ICAM in cells treated with LPS and HKEC (SupplementaryFigure 3; Supplementary Figure S7). ZO-1 expression increased in response to both HKEC and LPS in the scrambled (SCR) control cells (Supplementary Figure 3A; Supplementary Figure S7B). However, in the absence of GPNMB, ZO-1 expression remained unchanged, and the increase in expression in response to both inflammatory stimuli was abolished (Supplementary Figures S3A; 7B). While LPS enhanced ICAM expression in both SCR and siGPNMB cells, HKEC only affected ICAM expression in the SCR cells (Supplementary Figures S3B; 7C).

### GPNMB knockdown leads to enhanced proliferation but reduced migration

**Figure 4:**
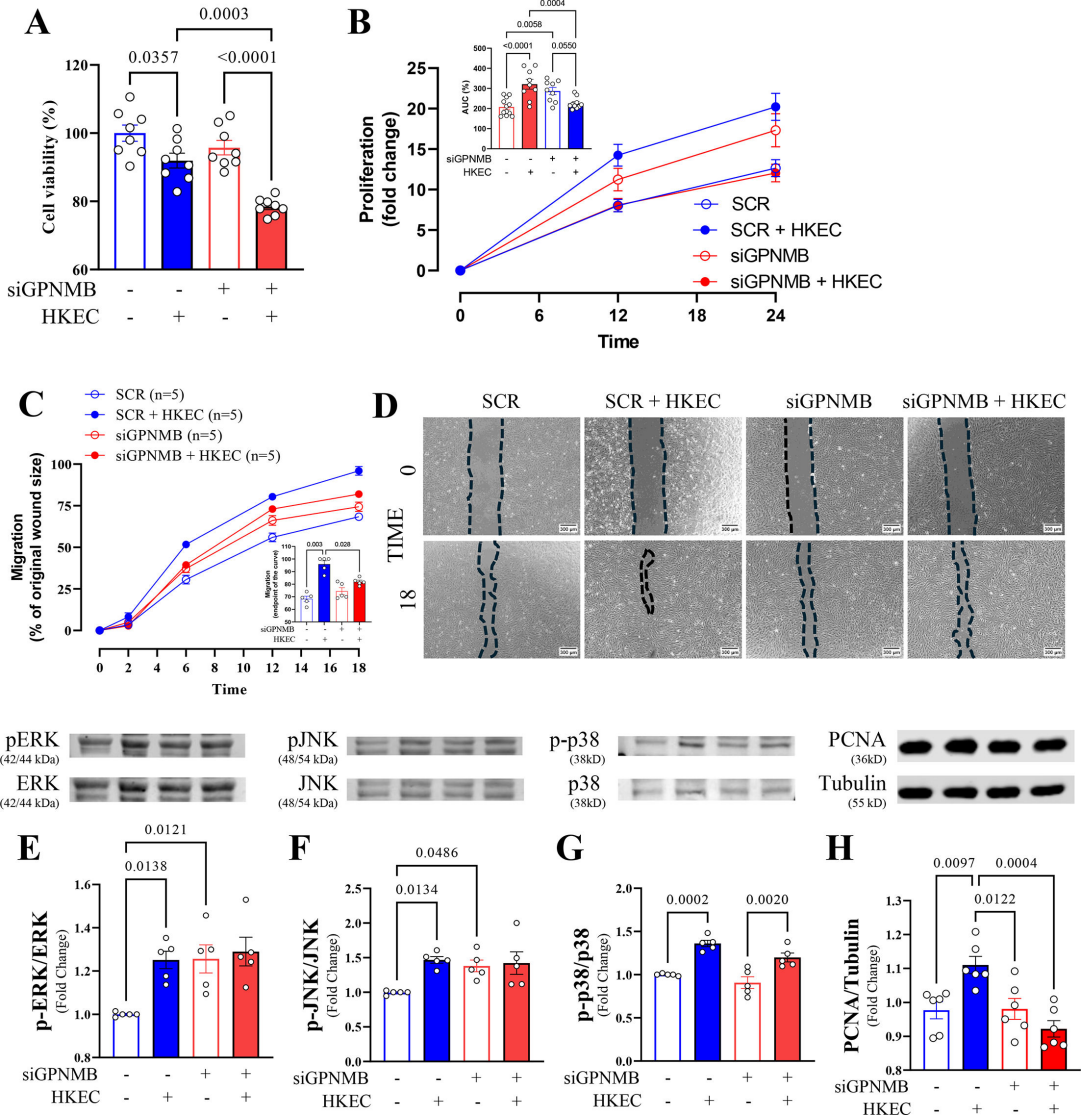
Effects of GPNMB-targeting siRNA on human microvascular endothelial cell viability, proliferation, and migration upon inflammatory challenge. Control HMVEC cells (SCR) and treated with siRNA for GPNMB silencing (siGPNMB) were stimulated with vehicle or heat-killed E. coli (HKEC, 3 × 10^8^ cells/ml) for 6 h. (**A**) Cell viability (CCK-8 assay) was assessed 1 h after the end of the entire protocol. (**B**) Proliferation (CCK-8 assay) was evaluated at 0-, 12-, and 24 h post-stimulation; the bar graph inserted in the figures represents the area under the curve (AUC) analysis of proliferation at 18 hours. (**C**) Cell migration was assessed using the Scratch-Wounding Assay at 2-, 6-, 12-, and 18 h post-stimulation; the bar graph inserted in the figures represents the quantification of the endpoint of the cell migration curve. (**D**) Representative images of the scratch-wounding assay at 0 and 18 h. (**E-G**) Phosphorylation levels of ERK, JNK, and p38 protein expression were normalized to their respective total protein levels. (**H**) PCNA protein expression normalized to α-Tubulin as a loading control. The number of samples used in each experiment (***n***) is expressed in parentheses or dots. Results are expressed as mean ± SEM. Statistics: Two-way ANOVA, followed by the Tukey post hoc test.

To better understand the relationship between GPNMB and tissue repair (cell growth and movement), we performed functional experiments to evaluate cell viability, migration, and proliferation after GPNMB knockdown. The reduction of GPNMB alone did not alter endothelial cells' viability; however, when exposed to HKEC, there was a significant reduction in cell viability in both siRNA-treated groups, but this reduction was of greater magnitude in siGPNMB cells than in control cells (Figure 4A). Alternatively, reducing GPNMB increased endothelial cell proliferation. However, when exposed to HKEC, control cells exhibited a significant increase in proliferation, whereas siGPNMB cells exposed to HKEC did not (Figure 4B). Regarding migration, decreased GPNMB expression without stimulation did not alter cell migration (Figure 4C and D). Conversely, exposure to HKEC promoted increased migration in control cells compared with siGPNMB cells. Subsequently, we focused on evaluating the expression of the mitogen-activated protein kinase (MAPK) superfamily, which is involved in intracellular signaling cascades that initiate various inflammatory cellular responses, such as regulating the production of inflammatory mediators, controlling cell proliferation, differentiation, migration, and survival of endothelial cells [6,36]. The relationship between phosphorylation and the total expression levels of ERK (Figure 4E; Supplementary Figure S5D) and JNK (Figure 4F; Supplementary Figure S5F) was enhanced in the presence of HKEC in SCR cells, indicating that these pathways are activated in response to inflammatory stimuli. However, in siGPNMB cells, both pERK (Figure 4E; Supplementary Figure S5C) and pJNK (Figure 4F; Supplementary Figure S5E) levels were elevated even without stimuli, and the presence of HKEC did not affect the expression of these proteins. The ratio of phosphorylated p38 mitogen-activated protein kinases (p-38) to total p38 expression was not altered by GPNMB knockdown; however, HKEC stimulation significantly increased p-38 phosphorylation in both groups (Figure 4G; Supplementary Figure S5A,B). The expression of proliferating cell nuclear antigen (PCNA), a reliable marker for assessing cell proliferation [37], increased in the presence of HKEC in SCR cells, although in siGPNMB cells, PCNA expression did not change in the absence or presence of HKEC (Figure 4H and Supplementary Figure S5C).

### GPNMB knockdown impedes the metabolic changes required for inflammatory responses

**Figure 5:**
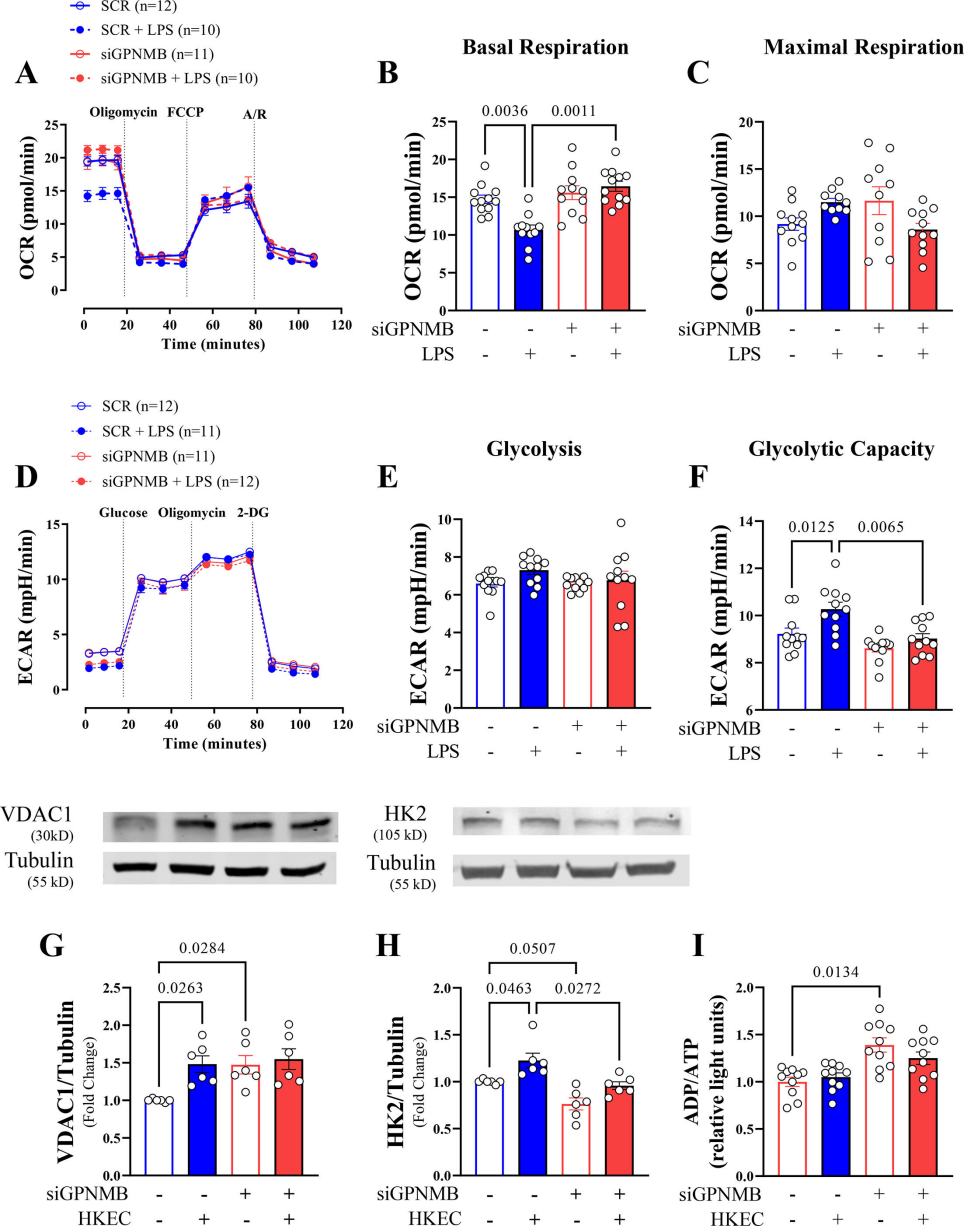
Metabolic profiling of HMVECs treated with control and GPNMB siRNA upon inflammatory challenge. Seahorse XFe96 assays for oxygen consumption rate (OCR, **A-C**) or extracellular acidification rate (ECAR; **D-F**) using Control and GPNMB siRNA-treated HMVECs with 6 h of LPS or HKEC exposure (100 ng/ml) prior to assays. (**A**) Respective components of OCR via calculation are as follows: (**B**) Basal respiration = pre Oligomycin – post Antimycin/Rotenone, (**C**) Max Respiration = post FCCP – post Antimycin/Rotenone. Respective ECAR components with calculations: (**E**) Glycolysis’ = post Glucose – pre Glucose, Glycolytic capacity = post oligomycin – post Glucose. *N* = 11–12 individual replicates per group. (**G-H**) VDAC1 and HK2 protein expression normalized to α-Tubulin as a loading control. (**I**) ADP/ATP ratio was measured in relative light units. The number of samples used in each experiment (***n***) is expressed in parentheses or dots. Results are expressed as mean ± SEM. Statistics: Two-way ANOVA, followed by the Tukey post hoc test.

Given the impact of reduced GPNMB expression on the inflammatory outputs examined above, we next wanted to test how GPNMB could alter endothelial metabolism, given the likely interdependence [38,39]. Metabolic networks are essential for energy production, biosynthesis, and redox regulation and are central pathways supporting cell proliferation. Using the Seahorse assay, we observed that the presence of GPNMB did not alter the basal oxygen consumption rate (OCR) but that in the presence of HKEC, there was a reduction only in control cells, with no change in siGPNMB (Figure 5A and B). When we evaluated maximal respiration (measured after stimulation with FCCP), no significant differences were observed between the groups (Figure 5A and C). Since voltage-dependent anion channel (VDAC) 1 is an essential regulator of mitochondrial function, we used its expression as a marker. VDAC1 expression was increased in siGPNMB EC. HKEC increased expression only in control ECs (Figure 5G; Supplementary Figure S6A,B). While VDAC1 is a mitochondrial marker, it has also been shown to regulate glycolytic metabolism through direct protein-protein interactions with hexokinase [40,41]. Thus, the extracellular acidification rate (ECAR) was used to monitor cellular glycolytic energy. However, glucose stimulation did not demonstrate a difference between the conditions evaluated (siGPNMB and HKEC) (Figure 5D-E). Instead, the application of oligomycin, which impairs mitochondrial respiration and shifts all energy production to glycolysis, showed that in the presence of HKEC, there was an increase in glycolytic capacity in control cells that was not observed in siGPNMB endothelial cells exposed to HKEC (Figure 5F). We then examined the expression of hexokinase 2 (HK2), the principal rate-limiting enzyme in the aerobic glycolysis pathway. HK2 expression was reduced in siGPNMB ECs, and as occurred with glycolytic capacity, HKEC only increased HK2 expression in control cells (Figure 5H; Supplementary Figure S6C-D). The metabolic changes caused by the GPNMB knockdown were confirmed by an increase in the ADP/ATP ratio (*P*<0.001), whereas stimulation with HKEC did not alter this condition (Figure 5I).

### Reintroduction of rGPNMB alters the ability of ECs to respond to the inflammatory response

For the rGPNMB reintroduction experiments, we chose concentration based on cell viability after 24 h of exposure to 10, 25, and 50 ng/ml of rGPNMB. The 50 ng/ml concentration reduced EC viability, so we used the 25 ng/ml concentration for the experiments. As mentioned previously (Figure 3D and Supplementary Figure S3B), the presence of HKEC increased ICAM expressions in SCR cells. This response was attenuated in siGPNMB cells (Figure 3D; Supplementary Figure S3B and D; Supplementary Figure S7C, Supplementary Figure S8B,E). In siGPNMB cells treated with rGPNMB, the presence of HKEC was not able to alter ICAM expression. Interestingly, the presence of rGPNMB increased the expression of Integrin B1 in endothelial cells, but this increase was not observed in cells that received rGPNMB and HKEC (Supplementary Figure 3E; Supplementary Figure 8C,F).

## Discussion

The host’s response to a severe infection, such as sepsis, depends on endothelial activation. This leads to increased blood flow and the recruitment of immune cells necessary for survival [24,42]. However, the host response is heterogeneous, leading to disparate outcomes. While multifactorial, proteins and cellular patterns related to a person’s age, or more specifically cellular senescence, likely contribute to that heterogeneity. Our study provides new insights into the role of GPNMB, a protein often linked to cellular aging, in endothelial cell function during infectious challenges and how its presence or absence regulates endothelial barrier integrity, cell proliferation, and metabolism. While our findings suggest that reducing GPNMB may provide protective effects under resting or homeostatic conditions, the presence of GPNMB during inflammatory stimuli is crucial in preventing endothelial cells from becoming dysfunctional. Thus, its role and regulation may have differential effects depending on the cellular stress and the endothelial cell environment. Beyond sepsis and infectious challenge, our observations also provide some insight into the contradictory impacts of GPNMB expression in models of inflammation. That role becomes particularly evident when we consider the reduced expression of GPNMB from patients experiencing septic shock, where multiple organ dysfunction occurs. In contrast, in patients with sepsis who do not have septic shock, the expression of GPNMB is similar to that seen in healthy individuals [26]. This suggests that GPNMB may be fundamental to the response and recovery after an infectious insult.

The host’s response to bacteria and their products promotes endothelial cell activation. This activation, in turn, allows them to serve as crucial participants in the immune response by releasing cytokines (IL-8 and TNF-α) and up-regulating adhesion molecules (ICAM and integrin) when stimulated by pathogens or inflammatory mediators [24,42–45]. Although primarily explored in macrophages owing to its high expression in that cell type, GPNMB has been implicated as a regulator of inflammation globally [16,17,46,47]. Whether it serves a similar, direct role in the endothelium is less well known, and this is the purpose of this study. The release of cytokines at lower levels and increased cellular barrier resistance in siGPNMB cells suggests that GPNMB knockdown conferred a protective effect, minimizing inflammatory responses under resting conditions. However, when challenged with bacteria, these cells (siGPNMB) failed to respond to inflammatory stimuli. This inability to respond to inflammation was characterized by a failure to increase adhesion molecule expression and a marked reduction in cellular barrier resistance. A decrease in GPNMB impaired endothelial cell activation capacity in response to stimuli. The inability to respond was observed in stimuli promoted by both LPS (toll-like receptor 4-dependent pathways) and HKEC (nonspecific pathways), suggesting its impact was not pattern recognition receptor-specific [48,49]. Instead, the role of GPNMB in modulating endothelial inflammatory responses is more universal. The reintroduction of rGPNMB yielded inconclusive results regarding its protective role against infectious stimulation in ECs. The observed increase in integrin β1 in cells treated with rGPNMB is likely due to the binding properties of rGPNMB [50,51]. The extracellular domain of GPNMB contains an RGD motif that binds to integrins, thereby helping maintain cell-cell adhesion [51,52]. However, integrin β1 expression decreased in cells treated with HKEC despite the presence of rGPNMB, suggesting that the effect is reversible. Additionally, ICAM-1 expression was attenuated in cells receiving rGPNMB when exposed to HKEC. Molecules like ICAM-1 are strongly regulated by inflammatory cytokines, suggesting that rGPNMB may significantly reduce the inflammatory response [53]. We did not find other studies that performed a rescue assay; however, under physiological conditions, rGPNMB (50 ng/ml) increased the expression of lymphangiogenic and autophagic markers in lymphatic endothelial cells [22]. Therefore, while endogenous GPNMB helps reduce inflammation, further experiments are needed to elucidate how reintroducing or overexpressing GPNMB influences the inflammatory response. Another essential mechanism involves the balance between matrix metalloproteinases (MMPs) and their inhibitors (TIMPs). MMPs disrupt tight junctions, and TIMP-1 protects them against this [33,54]. Reduced GPNMB expression significantly impaired TIMP-1 expression, increased MMP-2 activity, and, with this, further impaired their barrier function, further confirming the requirement of GPNMB for efficient inflammatory response.

Endothelial cell activation is crucial for the inflammatory response and tissue repair, as it regulates immune cell recruitment, vascular permeability, and the release of signaling molecules that promote healing and restore tissue integrity [55–58]. In control cells, stimulation with HKEC increased the phosphorylation of ERK and JNK, indicating that these kinases were activated in response to a bacterial challenge, likely as part of an inflammatory response. ERK and JNK can activate transcription factors such as AP-1 (c-Jun/Fos) and NF-κB, which then drive the expression of cell adhesion molecules [59–61]. These molecules are essential for recruiting immune cells and regulating the endothelial barrier during inflammation. In siGPNMB cells, basal phosphorylation levels of ERK and JNK were already elevated, suggesting that these pathways were in a ‘pre-activated state’ even before stimulation. Therefore, the transcriptional response required for the up-regulation of cell adhesion molecules was possibly impaired since HKEC did not further enhance ERK and JNK activation in siGPNMB cells [60,61]. The data suggest that these signaling pathways may have already reached a threshold or entered a feedback-inhibited state, preventing further activation upon bacterial stimulation. Appropriate inflammatory responses require dynamic changes in kinase activation rather than a constant, unregulated activation. GPNMB may act as a modulator that maintains ERK and JNK signaling responsiveness to external stimuli. Without GPNMB, ERK and JNK are trapped in a deregulated state, preventing the activation of essential transcription factors required to induce adhesion molecules. As a result, siGPNMB cells do not respond adequately to HKEC, leading to blunted expression of adhesion molecules and impaired proliferation and migration responses.

Many of the endothelium’s responses to infectious and inflammatory challenges are driven by the underlying metabolic state. Inflammatory activation of endothelial cells leads to a metabolic shift toward increased glycolysis with decreased mitochondrial respiration, correlating with the up-regulation of glycolytic enzymes, such as HK2 [10,38]. The data from the present study show that this metabolic shift is caused by HKEC stimulation; however, cells with reduced GPNMB expression do not have the same capacity to adjust metabolically. The involvement of GPNMB in metabolic responses has been previously evaluated in cancer cells, where, as in inflammation, metabolic reprogramming is crucial for cell signaling [50,62]. In cancer cells, GPNMB binds to its CD44 receptor on mesenchymal stem cells to promote glycolysis through the activation of Protein-tyrosine kinase 2 (Pyk2) [50,62,63]. Additionally, some studies demonstrate that Pyk2 plays an essential role in activating MAPKs [62–65]. Thus, we demonstrate that the absence of GPNMB affects the ability of these cells to reprogram metabolic metabolism and respond efficiently to infectious challenges. This deficiency in metabolic responses observed in siGPNMB cells was confirmed by the increased ADP/ATP ratio. When considered alongside other data on endothelial activation after infectious challenges, this observation highlights the essential role of GPNMB in regulating the cellular shift in metabolic phenotype. Thus, in endothelial cells lacking GPNMB, metabolism is disrupted and lacks the capacity to generate the rapid energy required for cell survival. This suggests that GPNMB is at the crossroads of metabolically driven endothelial structure and function during acute infections and may serve as a target when disturbances in these pathways result in a harmful vascular phenotype in patients facing a severe pathogen-induced host response.

This study has limitations, particularly the in vitro application of bacteria and bacterial toxins to isolated endothelial cells to investigate the role of GPNMB in endothelial signaling. Although *in vivo* models are physiologically relevant, they involve complex interactions among multiple cell types and systemic factors, which makes it challenging to attribute effects to endothelial cells alone—particularly since GPNMB is expressed in diverse cell types and exists in both soluble and membrane-bound forms. Our primary observations of GPNMB changes in human systems exposed to bacteria (e.g. septic patients and ex vivo stimulation) are based on PBMCs and leukocytes, which are more readily available from humans with pathological conditions such as sepsis. However, they may not reflect what is happening in vascular tissue under the same conditions. Further, we opted to use dermal endothelial cells because the skin is one of the primary clinical sources for assessing perfusion and tissue edema. Yet, the endothelium is heterogeneous and often tissue-specific, so the impact of GPNMB in regulating the endothelial response to bacterial stimulation may be unique to the vascular bed being tested. Despite these limitations, *in vitro* analysis based on clinical observations provides a controlled environment and enables precise molecular manipulations, thereby yielding clear mechanistic insights that complement and inform future *in vivo* investigations. To understand a broader role of GPNMB in immune cells, particularly macrophages and lymphocytes, further examination should be performed in whole animal models to investigate how GPNMB influences immune cell function and outcomes during sepsis.

## Conclusion

Overall, our findings highlight the vital and multifaceted role of GPNMB in endothelial cells. While the GPNMB knockdown reduces inflammation and reinforces baseline barrier integrity, it also increases the risk of barrier disruption, impairs endothelial migration, and alters metabolic responses to inflammatory triggers. These findings position GPNMB as a key regulator of endothelial homeostasis and inflammation, suggesting its significant relevance in addressing vascular diseases associated with endothelial dysfunction in the context of bacterial challenge.

**Clinical Perspectives**

- Severe infections, such as sepsis, activate endothelial cells, crucial for blood flow and immune cell recruitment. Host responses vary widely and are influenced by factors such as cellular senescence. Glycoprotein nonmetastatic melanoma protein B (GPNMB), associated with cellular aging, lacks clarity in its role in endothelial function during infections. Understanding GPNMB's impact on endothelial cells can reveal mechanisms behind varied host responses.
- GPNMB is down-regulated in sepsis and blood samples are exposed to bacterial pathogens. Knockdown of GPNMB in endothelial cells altered gene expression, leading to anti-inflammatory effects, such as decreased IL-8 and TNFα levels during bacterial stimulation. It improved barrier function under normal conditions but increased susceptibility to disruption during inflammation. Additionally, when exposed to bacteria, GPNMB knockdown increased cell proliferation but decreased migration and viability, disrupting metabolic responses and energy production essential for inflammatory processes.
- GPNMB is a critical regulator of inflammatory diseases and vascular dysfunction, highlighting its potential as a therapeutic target.

## Supplementary material

online supplementary figure 1.

online supplementary figure 2.

online supplementary figure 3.

online supplementary figure 4.

online supplementary figure 5.

online supplementary figure 6.

online supplementary figure 7.

online supplementary figure 8.

online supplementary table 1.

## Data Availability

The datasets generated and/or analyzed during the current study are publicly available in the Gene Expression Omnibus (GEO) database. The primary dataset generated for this study is available under the accession number GSE295012 and can be accessed at https://www.ncbi.nlm.nih.gov/geo/query/acc.cgi?acc=GSE295012.GSE295012 The Loss of Glycoprotein Nonmetastatic Melanoma Protein B (GPNMB) Worsens Endothelial Cell Permeability, Metabolism, and Survival During Infectious Challenge Jan 01, 2027 [66]. GSM8940100 siControl imHMVECs 1 Jan 01, 2027 GSM8940101 siControl imHMVECs 2 Jan 01, 2027 GSM8940102 siControl imHMVECs 3 Jan 01, 2027 GSM8940103 siGPNMB imHMVECs 1 Jan 01, 2027 GSM8940104 siGPNMB imHMVECs 2 Jan 01, 2027 GSM8940105 siGPNMB imHMVECs 3 Jan 01, 2027

## Abbreviations

ECAR: Extracellular acidification rate
GEO: Gene Expression Omnibus
GPNMB: glycoprotein nonmetastatic melanoma protein B
HK2: Hexokinase 2
HKEC: heat-killed *Escherichia coli*
HKSA: heat-killed S. aureus
HMVEC: Human dermal microvascular endothelial cells
ICAM-1: Intercellular Adhesion Molecule 1
LPS: Lipopolysaccharide
MMP: Matrix metalloproteinase
OCR: Oxygen consumption rate
TEER: Transendothelial electrical resistance
VDAC1: voltage-dependent anion channel 1
