# Supplementary figures and images for: The loss of glycoprotein nonmetastatic melanoma protein B (GPNMB) alters endothelial cell permeability, metabolism, and survival during infectious challenge
Source: Clin Sci (Lond). 2026 Jan 14;140(1):115–30. doi: 10.1042/CS20256682 (PMC12862960; doi:10.1042/CS20256682)

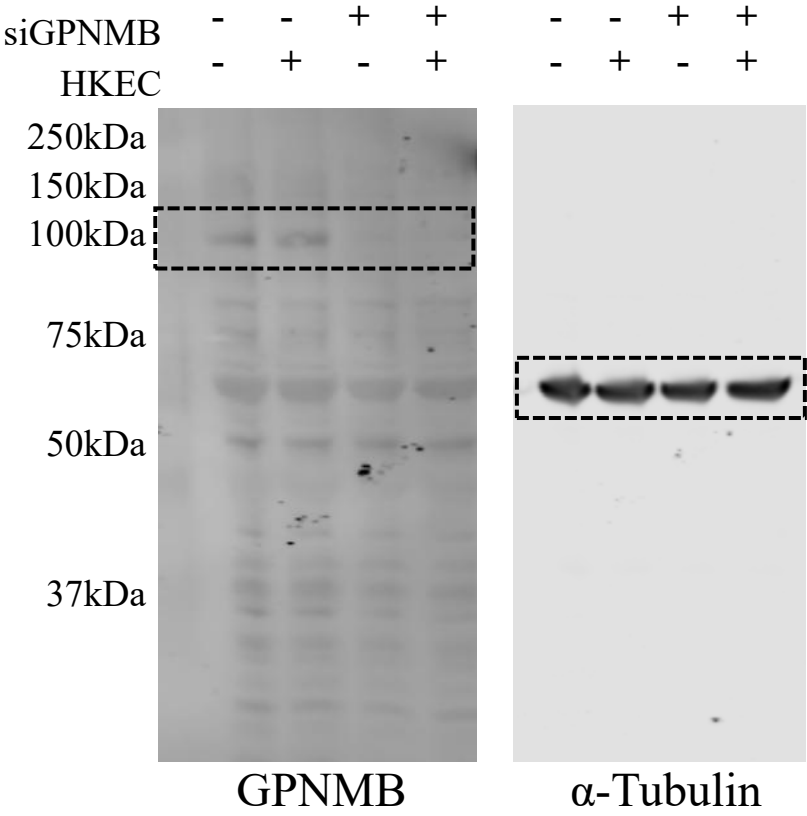

Supplemental Figure 1.

Supplement: online supplementary figure 1. [file cs-140-1-CS20256682-s001.pdf]

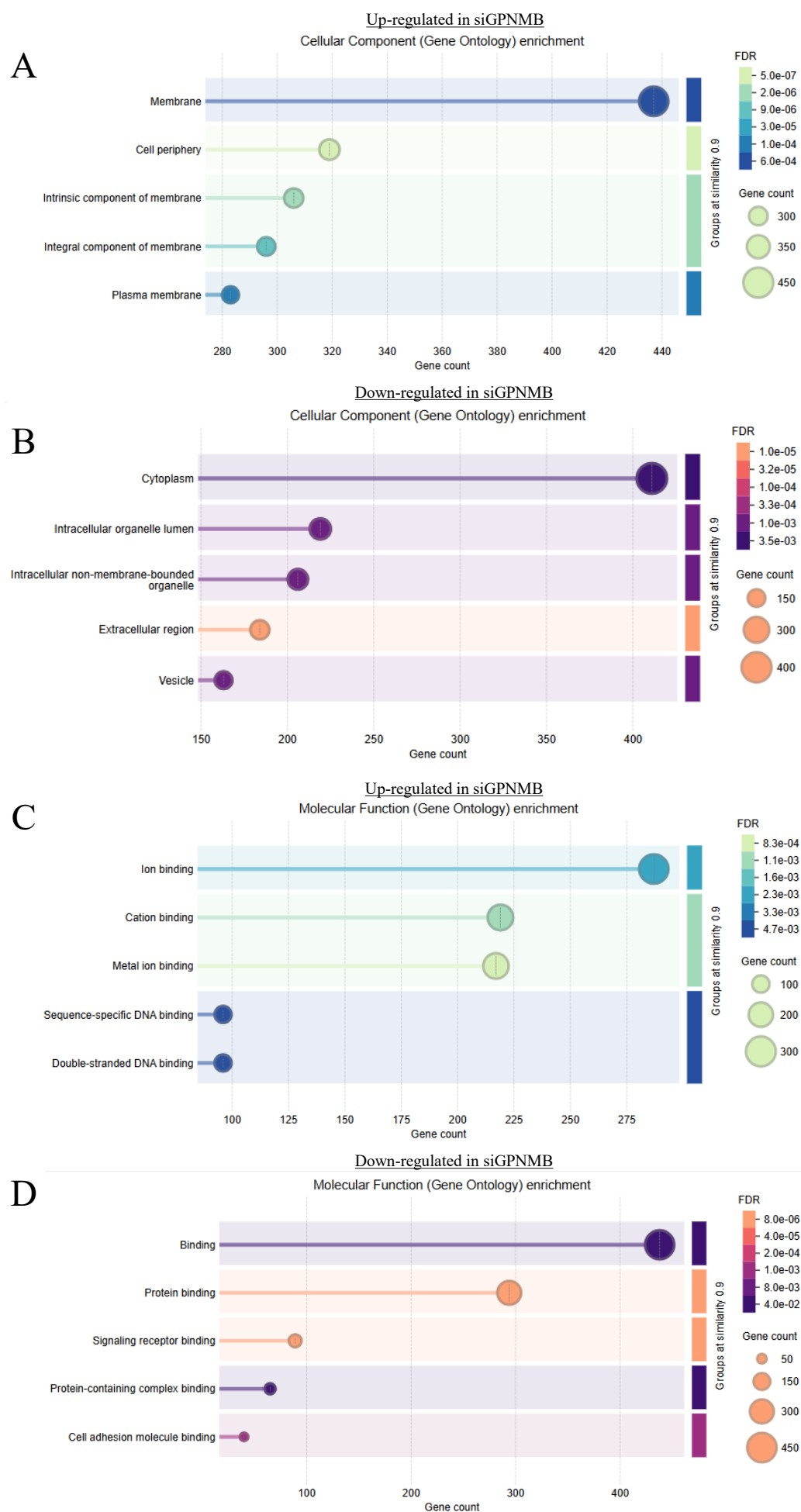

Supplemental Figure 2.

Supplement: online supplementary figure 2. [file cs-140-1-CS20256682-s008.pdf]

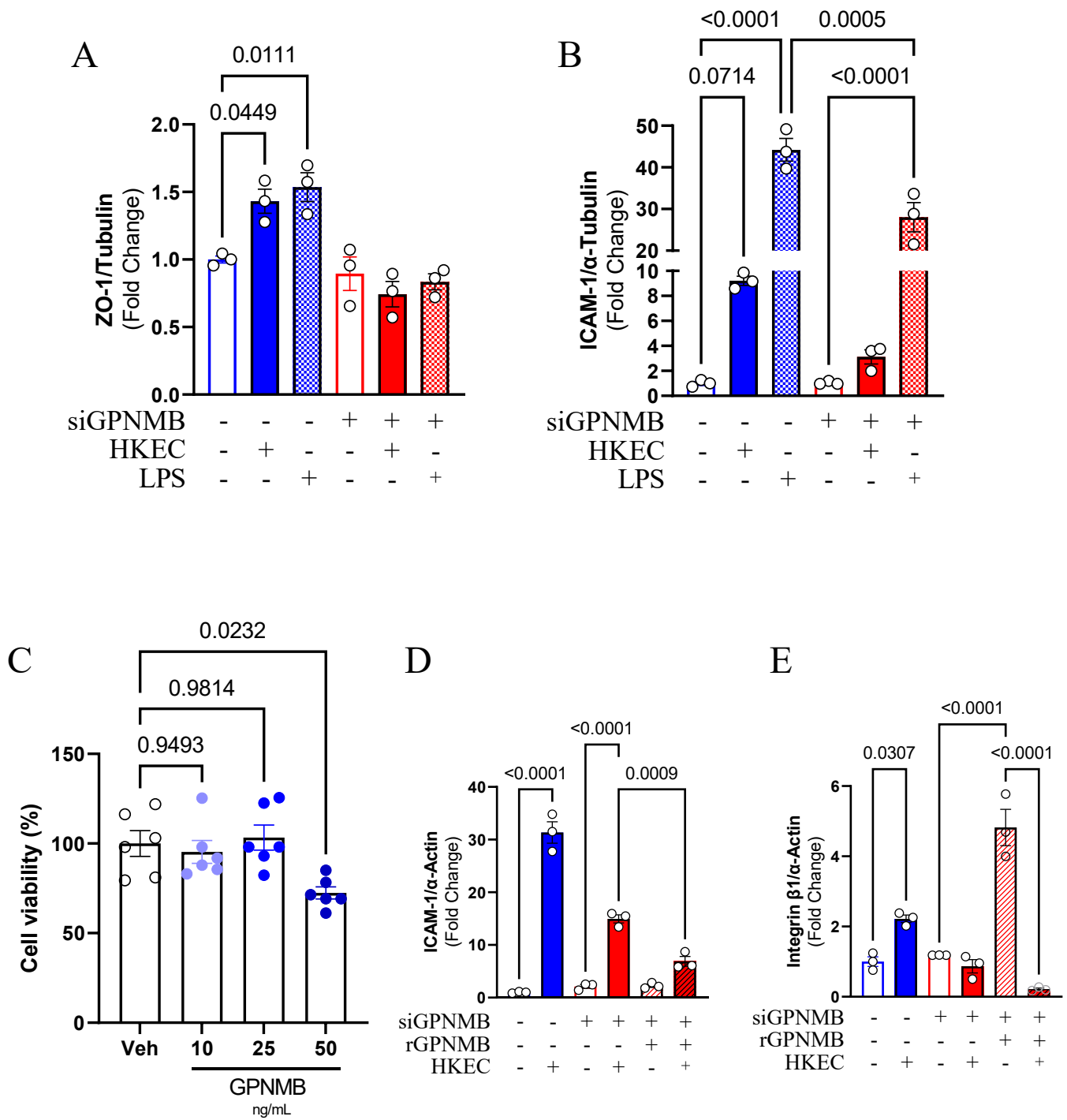

Supplemental Figure 3.

Supplement: online supplementary figure 3. [file cs-140-1-CS20256682-s003.pdf]

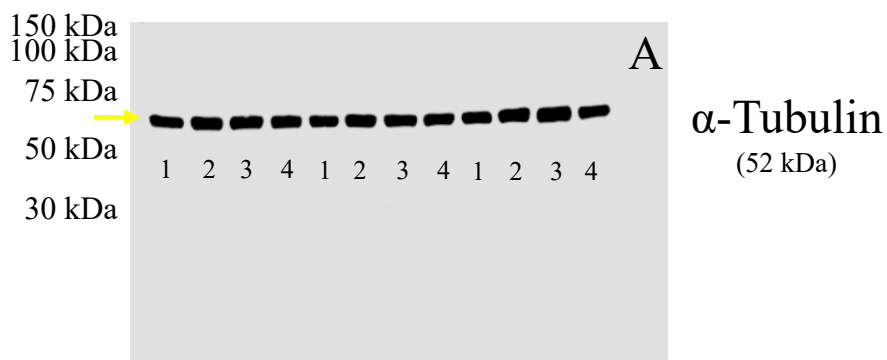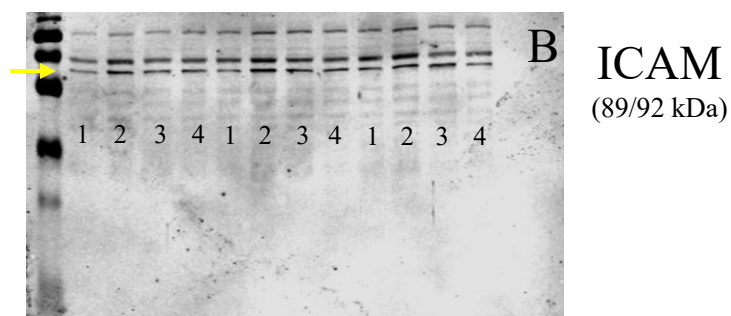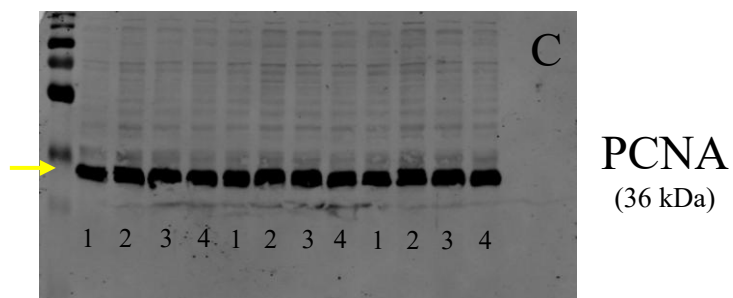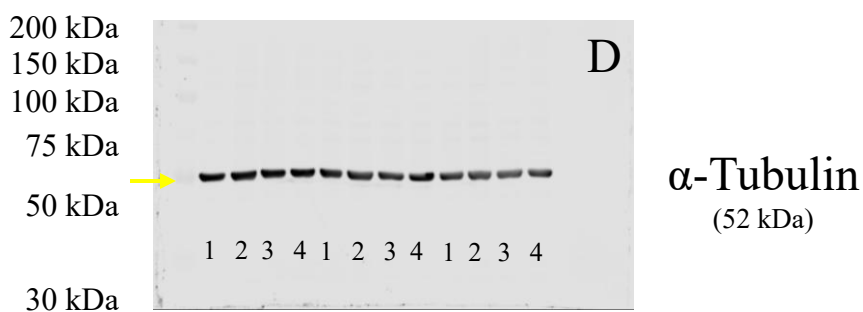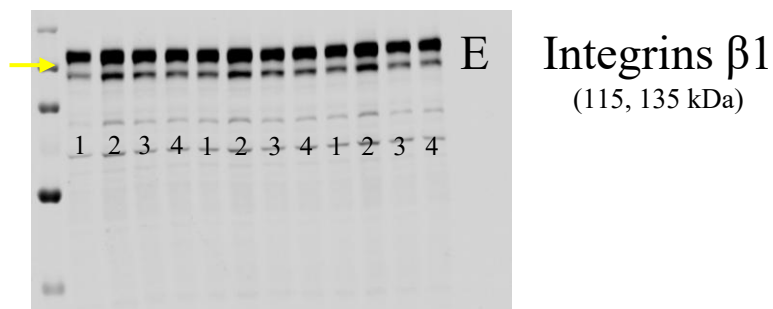

1. SCR
2. SCR + HKEC
3. siGPBMB
4. siGPBMB + HKEC

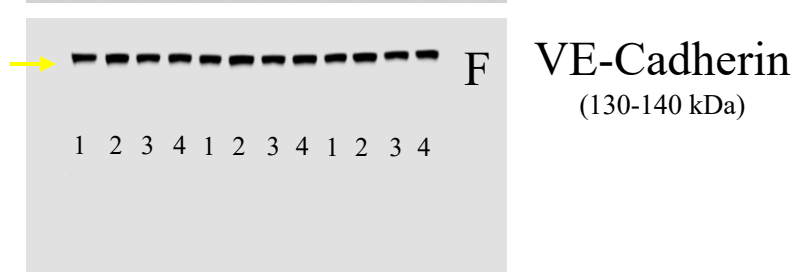

Supplemental Figure 4.

Supplement: online supplementary figure 4. [file cs-140-1-CS20256682-s002.pdf]

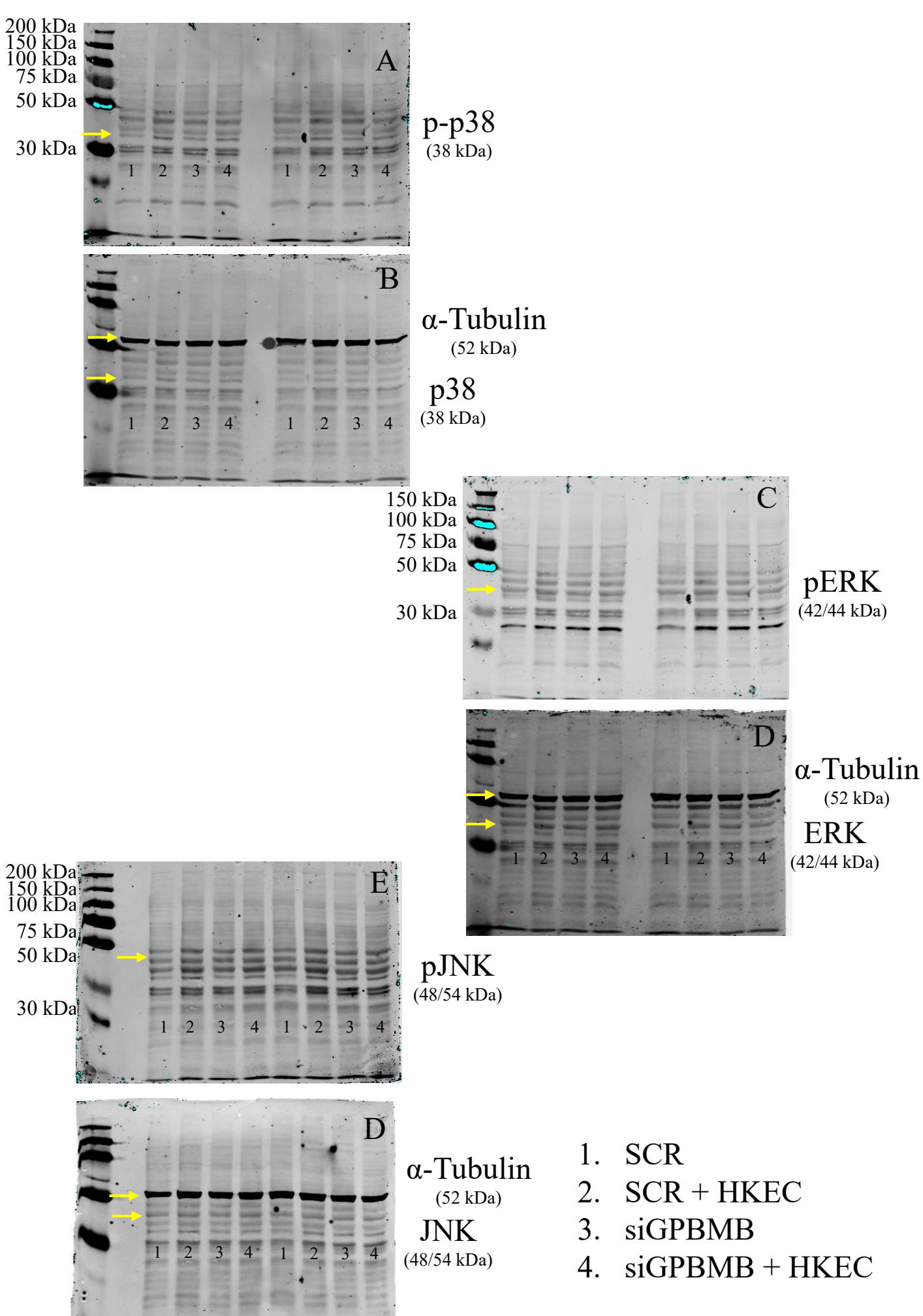

Supplemental Figure 5.

Supplement: online supplementary figure 5. [file cs-140-1-CS20256682-s005.pdf]

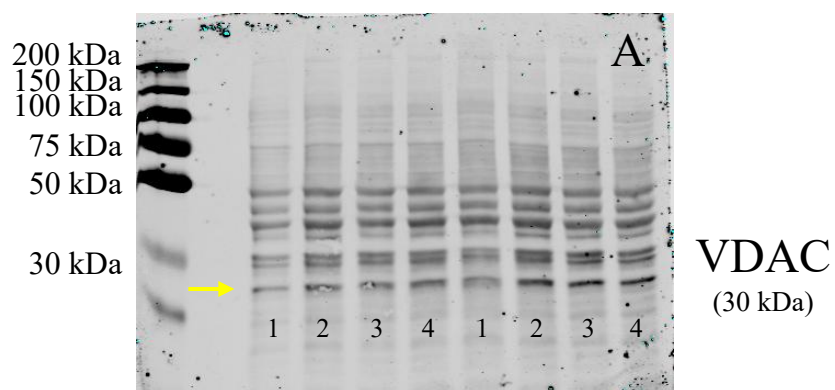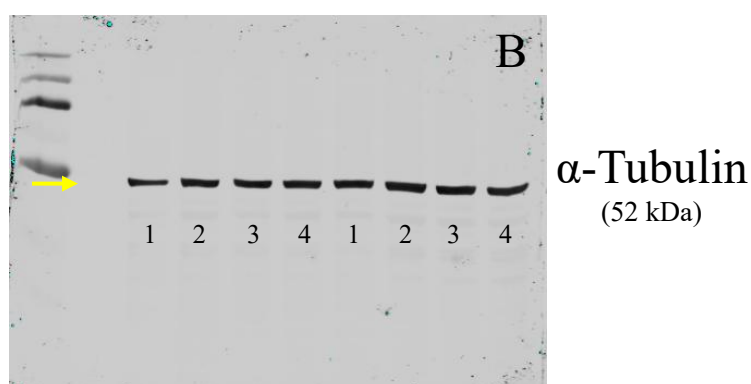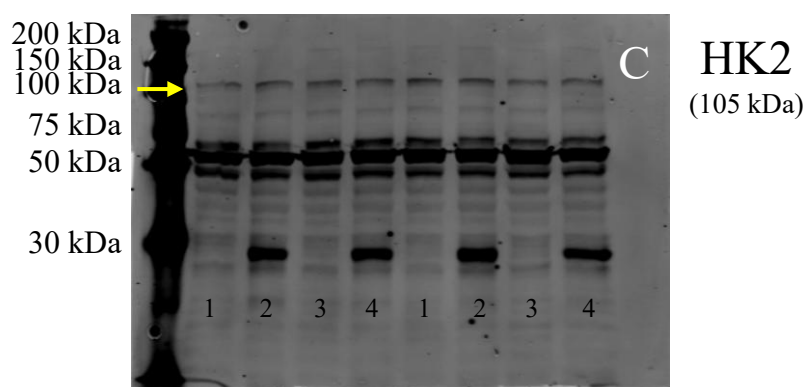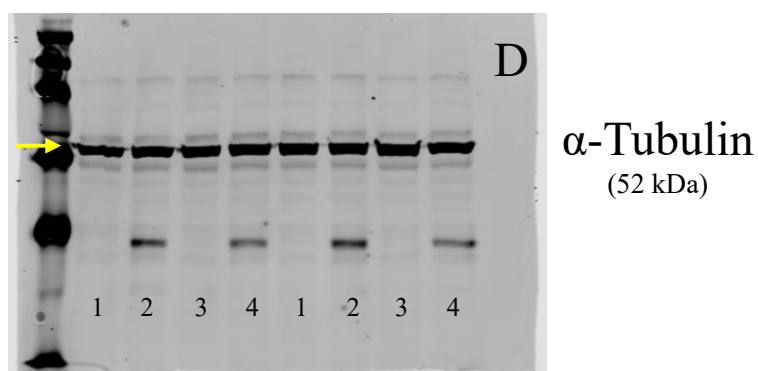

1. SCR
2. SCR + HKEC
3. siGPBMB
4. siGPBMB + HKEC

Supplemental Figure 6.

Supplement: online supplementary figure 6. [file cs-140-1-CS20256682-s006.pdf]

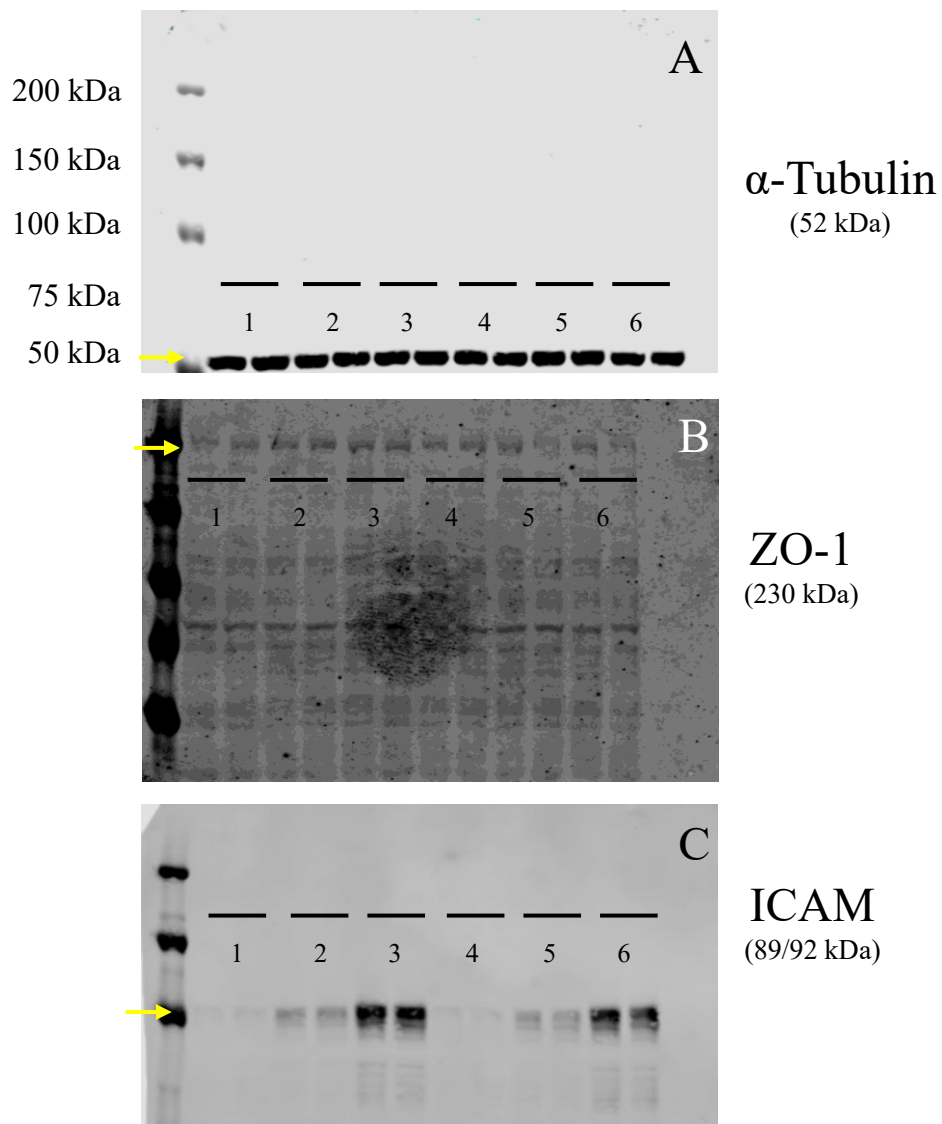

1. SCR
2. SCR + HKEC
3. SCR + LPS
4. siGPBMB
5. siGPBMB + HKEC
6. siGPBMB + LPS

Supplement: online supplementary figure 7. [file cs-140-1-CS20256682-s004.pdf]

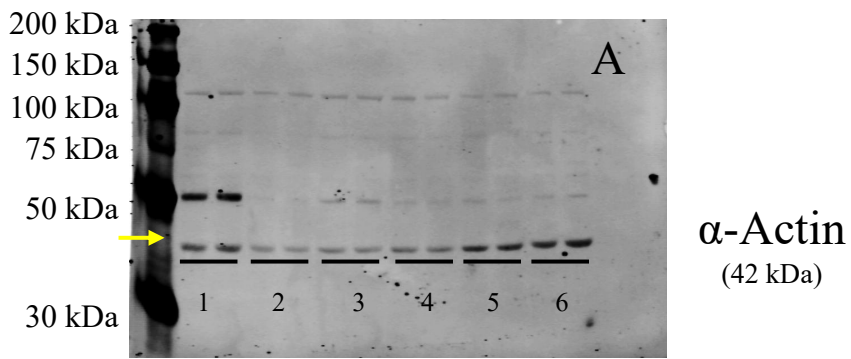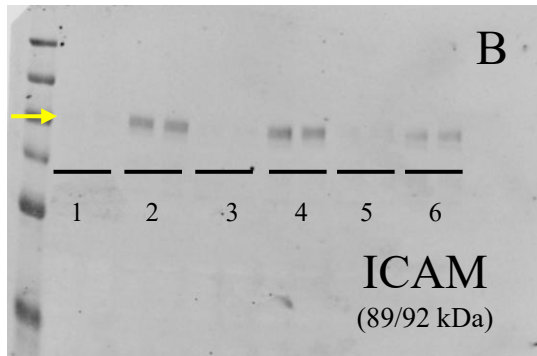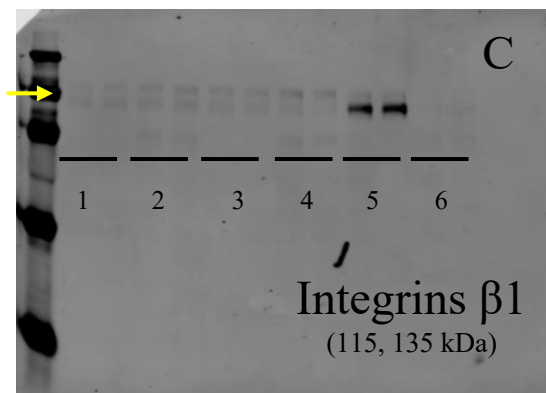

1. SCR
2. SCR + HKEC
3. siGPBMB
4. siGPBMB + HKEC
5. siGPBMB + GPNMB
6. siGPBMB + GPNMB + HKEC

Supplement: online supplementary figure 8. [file cs-140-1-CS20256682-s007.pdf]
