# Supplementary material for: The loss of glycoprotein nonmetastatic melanoma protein B (GPNMB) alters endothelial cell permeability, metabolism, and survival during infectious challenge
Source: Clin Sci (Lond). 2026 Jan 14;140(1):115–30. doi: 10.1042/CS20256682 (PMC12862960; doi:10.1042/CS20256682)
Supplement: online supplementary table 1. [file cs-140-1-CS20256682-s009.docx]

**Supplemental Table 1.** Antibodies used in Western blotting experiments.

| Antibody | Catalog | Supplier | Dilution |
| --- | --- | --- | --- |
| p38 MAPK Rabbit | 9212S | Cell Signaling Technology | 1:1000 |
| Phospho-p38 MAPK (Thr180/Tyr182) Mouse | 9216S | Cell Signaling Technology | 1:1000 |
| SAPK/JNK Rabbit | 9252S | Cell Signaling Technology | 1:1000 |
| Phospho-SAPK/JNK (Thr183/Tyr185) (G9) Mouse mAb | 9255S | Cell Signaling Technology | 1:1000 |
| p44/42 MAPK (Erk1/2) Rabbit | 9102S | Cell Signaling Technology | 1:1000 |
| Phospho-p44/42 MAPK (Erk1/2) (Thr202/Tyr204) Rabbit | 9101S | Cell Signaling Technology | 1:1000 |
| Hexokinase II (C64G5) Rabbit mAb | 2867S | Cell Signaling Technology |  |
| VDAC Rabbit mAb | 4661S | Cell Signaling Technology | 1:1000 |
| VE-Cadherin (D87F2) XP® Rabbit mAb | 2500S | Cell Signaling Technology | 1:5000 |
| α-Tubulin (DM1A) Mouse mAb | 3873S | Cell Signaling Technology | 1:15000 |
| PCNA | 05-347 | Upstate | 1:500 |
| Integrin β1 (D6S1W) Rabbit mAb | 34971S | Cell Signaling Technology | 1:1000 |
| CD54/ICAM-1 Rabbit | 4915S | Cell Signaling Technology | 1:1000 |
| Alpha smooth muscle actin Mouse | 67735-1 | Proteintech | 1:10000 |
